# The Senescence-Related Signature Predicts Prognosis and Characterization of Tumor Microenvironment Infiltration in Pancreatic Cancer

**DOI:** 10.1155/2022/1916787

**Published:** 2022-12-05

**Authors:** Hao Hua, Chenglong Zheng, Jiling Fan, Xiushen Li, Wenfeng Xie, Jinxin Chen, Chao Yu

**Affiliations:** ^1^Department of Hepatic-Biliary-Pancreatic Surgery, The Affiliated Hospital of Guizhou Medical University, Guiyang, Guizhou, China; ^2^Department of Hepatobiliary Surgery, Shenzhen Key Laboratory, Shenzhen University General Hospital, Shenzhen, Guangdong, China; ^3^Shenzhen University, Shenzhen, Guangdong, China; ^4^Department of Obstetrics and Gynecology, Shenzhen Key Laboratory, Shenzhen University General Hospital, Shenzhen, Guangdong, China

## Abstract

**Background:**

Senescence is thought to be an imperative effect on the development of cancer. However, few studies pay an attention to the senescence-associated genes in pancreatic cancer (PC). The prognostic value of senescence-related genes (SRGs) and their involvement in tumor microenvironment (TME) in the PC remain obscure. The aim of this research was to investigate the prognostic role of senescence-associated genes and their affection in TME in PC.

**Methods:**

The transcriptome and clinical information of PC patients were obtained from The Cancer Genome Atlas (TCGA) and the Gene Expression Omnibus (GEO) databases. Two SRG-mediated molecular clusters were comprehensively identified. In total, data from the 285 PC patients were randomly used to develop a senescence-associated gene signature in the training set and verified in the validation set. Immune microenvironment analysis pertained to senescence-related genes was performed.

**Results:**

A SRG_score including five senescence-associated genes was established to separate PC patients into two risk groups. High-risk patients had worse overall survival than low-risk patients. The result of the multivariate Cox regression analysis identified the risk score and stage as independent prognostic factors for PC patients. Receiver operating characteristic curve (ROC) analysis confirmed the credible predictive ability of the nomogram. The area under time-dependent ROC curve (AUC) reached 0.746 at 1 year, 0.781 at 3 years, and 0.868 at 5 years in the training set and 0.653 at 1 year, 0.755 at 3 years, and 0.785 at 5 years in the validation set. Moreover, the SRG_score was associated with TME, tumor mutation burden (TMB), and chemotherapeutic drug sensitivity.

**Conclusions:**

This study found that the novel SRG_score could be an independent prognostic target for PC patients. Senescence-associated genes had a vital impact on the immune microenvironment and the treatment of PC patients.

## 1. Introduction

Pancreatic cancer (PC) is one of the most malignant tumors. According to the statistics of the National Cancer Association, the mortality rate of the PC ranks fourth, and the five-year survival rate is less than 10% [1]. Moreover, there are no specific manifestations in early PC, which is mainly characterized by abdominal pain, jaundice, gastrointestinal symptoms, weight loss, and fatigue. Abdominal mass may occur in the middle and late stages. Traditional therapy methods, including surgery, chemotherapy, and radiotherapy, have a poor effect on the prognosis. In recent years, immunotherapy has become a hot spot in tumor therapy, which brings new hope for the patients [2]; however, immunotherapy has not yet reached the desired effect in the treatment of pancreatic cancer. Although there has been continuous research on the diagnosis and treatment methods of PC for many years, it is found that the long-term survival rate of PC patients has not improved significantly, so researchers began to explore different areas.

The biological and therapeutic response of PC is further shaped by numerous forms of regulated cell death, such as apoptosis, necroptosis, ferroptosis, senescence, and alkaliptosis. Among them, senescence serves a key role in the mechanism of tumor and is characterized by cell cycle arrest, aging-related phenotype, macromolecular damage, and metabolic disorder [3]. Nowadays, a great number of studies have concluded a close relationship between tumors and senescence [4, 5]. Most tumors occur in the aging period of the body, and senescent cells are difficult to repair mismatch genes, which are more likely to lead to the activation of oncogenic genes and the inactivation of tumor suppressor genes [6]. Cellular senescence refers to the process that is mainly manifested in the decline of the ability of cell proliferation and differentiation and physiological function with the passage of time [7, 8]. Cellular senescence can affect the therapeutic effect of the tumor by the mechanism of cell autonomous and cell nonautonomous [9]. It is worth noting that due to the specific physiological environment of tumor cells and the complex environment of the organism, the mechanisms of tumor cell autonomous and nonautonomous show a variety of biological effects on the regulation of cellular senescence, having a comprehensive impact on the occurrence of tumor and the effect of chemotherapy [10], and can lead to two opposite effects of tumor promotion or tumor inhibition [11]. Recent study has found that complement factor B (CFB) could promote proliferation by preventing cellular senescence and had profound implication in immunological tumor promotion in PC [12]. A growing body of studies elucidated that senescence is dependent of its role in the proliferation and migration of PC cells and has been demonstrated to play an irreplaceable role in promoting inflammatory cell death of PC [13, 14].

Tumor immune microenvironment (TME) is a complicated and evolving environment and has the advantage of guiding tumor progression through manipulating immune functions [15]. Previous evidence also demonstrated that the induced senescence is correlated with immune cell intrinsic and extrinsic factors from the tumor immune microenvironment [16]. Accumulating studies suggested that senescence of T cell populations plays an important role in promoting cancer particularly [17, 18]. Implanted with preneoplastic skin, breast, and prostate cell lines of mouse and human origins with senescent fibroblasts, the growth of the tumor is more obvious compared to these cells without the senescent fibroblasts [19, 20]. In the TME, senescent tumor cells may increase antitumor immune responses by secreting IL-6, IL-8, and insulin-like growth factor binding protein 7 (IGFBP7) with the ability of recruiting immune cells such as T lymphocytes to the tumor site [21]. Above all, it has great research value for deepening the function of senescence in TME, tumor growth, and proliferation and developing more effective anticancer drugs. However, our understanding of the effect of senescence in the pathogenesis and TME of PC is still limited. More and more predictive models are used to predict the prognosis of cancer patients [22]. Hence, with the development of a molecular subtype classification pattern based on senescence-related genes (SRGs) and the features of TME cell infiltration modulated by multiple SRGs, it is beneficial to understand the mechanism of PC oncogenesis and predict the response to the effect of potent anticancer drugs targeting molecules.

## 2. Materials and Methods

### 2.1. Data Acquisition

We obtained the transcriptome data of PC patients from The Cancer Genome Atlas (TCGA) database (https://portal.gdc.cancer.gov/) and the gene expression files (GSE57495 from the GPL15048 platform and GSE62452 from the GPL2644) from the Gene Expression Omnibus (GEO) (https://www.ncbi.nlm.nih.gov/geo/). The inclusion criteria were as follows: (1) available for overall survival (OS) of data and senescence-related mRNA expression; (2) diagnosed as PC; and (3) follow-up data. We excluded PC patients without detailed follow-ups. The fragments per kilobase of transcript per million (FPKM) values of TCGA data were changed into transcripts per kilobase million (TPM) values through performing the R package “limma.” The transcriptome data were transformed to the format of Log2[transcripts per million (TPM) + 1]. To merge the two datasets and eliminate batch effects, we used the combat algorithm included in the SVA R package.

### 2.2. Prognostic- and Senescence-Related Gene Cluster Analysis and Relationship between Molecular Subtypes with the Prognostic of PC

Seventy-seven SRGs were obtained via screening the MSigDB Team (http://www.broad.mit.edu/gsea/msigdb/). Among these genes, genes with a *p* value < 0.05 were considered prognostic- and senescence-related genes (SRGs) via the univariate Cox regression analysis. According to prognostic-SRG expression, we applied the consensus unsupervised clustering analysis function of the R package “ConsensusClusterPlus” to develop a distinct senescence-related molecular subtype for further analysis. PC samples were distinguished, and the consensus clustering algorithm was subjected to adjust the stability and patterns of molecular subtypes. Gene set variation analysis (GSVA) was utilized to explore the differences in the biological procession of SRGs. Furthermore, the Kaplan-Meier curve was generated to analyze the prognostic difference between different subtypes by the “survival” and “survminer” R packages.

### 2.3. Identification of Differentially Expressed Genes among Subtypes and Functional Annotation

To identify DEGs between the two senescence subtypes, we performed the “limma” package in R with the significance criteria of a fold-change of 2 and an adjusted *p* value of < 0.05. The Gene Ontology (GO) analysis and the Kyoto Encyclopedia of Genes and Genomes (KEGG) pathway analysis were executed to identify the related gene functions and enriched pathways using the “clusterprofiler” package in R.

### 2.4. Correlations of Molecular Subtypes with TME in PC

The ESTIMATE algorithm was performed to investigate the immune and stromal levels of each patient. The abundance of 23 immune cell types based on all gene expression levels was calculated by the CIBERSORT algorithm, and we applied a single-sample gene set enrichment analysis (ssGSEA) algorithm to investigate the expression of immune cell infiltration in the PC TME.

### 2.5. Establishment of the Senescence-Related Prognostic SRG_model

The senescence-related prognostic SRG_model was constructed. First, the DEGs associated to PC were entered into the univariate Cox regression analysis for identifying the OS. Second, according to the expression of prognostic SRGs, different subtype groups (senescence gene subtype A, senescence gene subtype B, and senescence gene subtype C) were generated among patients via an unsupervised clustering method. Finally, all PC patients were randomly assigned to either training set or validation set. In other words, according to the prognostic- and senescence-related genes, the absolute shrinkage and selection operator (LASSO) penalty for analysis and lowest Akaike information criterion (AIC) value were applied to identify the best prognostic value of these genes. The genes screened out via this method were further used to establish a prognostic SRG_model in the training set. The formula of risk score was as follows: e^sum^ (normalized expression level of each senescence − associated gene × corresponding regression, where Coefi is the coefficient from the multivariate Cox regression analysis and Expi is the relative expression of each gene, respectively. Based on the optimal cutoff value of risk score by using the “survminer” R package, a total of PC patients in the training set were divided into low-risk (SRG_score < median value) and high-risk (SRG_score > median value) groups and then explored the prognostic value of this system by the Kaplan-Meier survival analysis. As previously provided, the testing set was categorized into low- and high-risk groups, and the prognostic value of patients in these two risk groups was compared using the similar method of the R “survival” package. Then, time-dependent receiver operating characteristic (ROC) curves for 1-, 3-, and 5-year survival were used to evaluate the predictive accuracy of the SRG_score in the two sets.

### 2.6. Construction and Validation of a Nomogram Scoring System

A predictive nomogram was developed to predict three different years of survival ratio based on the outcome of the risk score and disease stage using the R package “rms.” Calibration plots were used to describe the predictive value between the predicted 1-, 3-, and 5-year survival outcomes and the actual observations.

### 2.7. Evaluation of Immune Status between the Two Different Risk Groups

To evaluate the proportions of tumor-infiltrating immune cells (TIICs), CIBERSORT was applied to quantify the abundance of 23 infiltrating immune cells in heterogeneous samples in the low- and high-risk groups. We also used box plots to examine the differential expression levels of 23 infiltrating immune cells among the three gene cluster groups. Meanwhile, we also investigated the correlations between SRG_score and infiltrating immune cells.

### 2.8. Mutation and Drug Susceptibility Analysis

The somatic mutation data of PC patient from the TCGA database was depicted using the “maftools” R package. We also compared the tumor mutation burden (TMB) score between high- and low-risk groups. We also explored the semi-inhibitory concentration (IC_50_) values of chemotherapeutic drugs, including multitarget kinase inhibitors, DNA synthesis inhibitors, and immunomodulators, to compare the sensitivity to several chemotherapy drugs related to the selected risk signature genes by the R “pRRohetic” package [23].

### 2.9. Statistical Analyses

R software (version 4.1.0) was performed in all statistical analyses. A *p* < 0.05 was regarded statistically significant, and all *p* values were two tailed.

## 3. Results

### 3.1. The Expression Level of SRGs in PC

**Figure 1:**
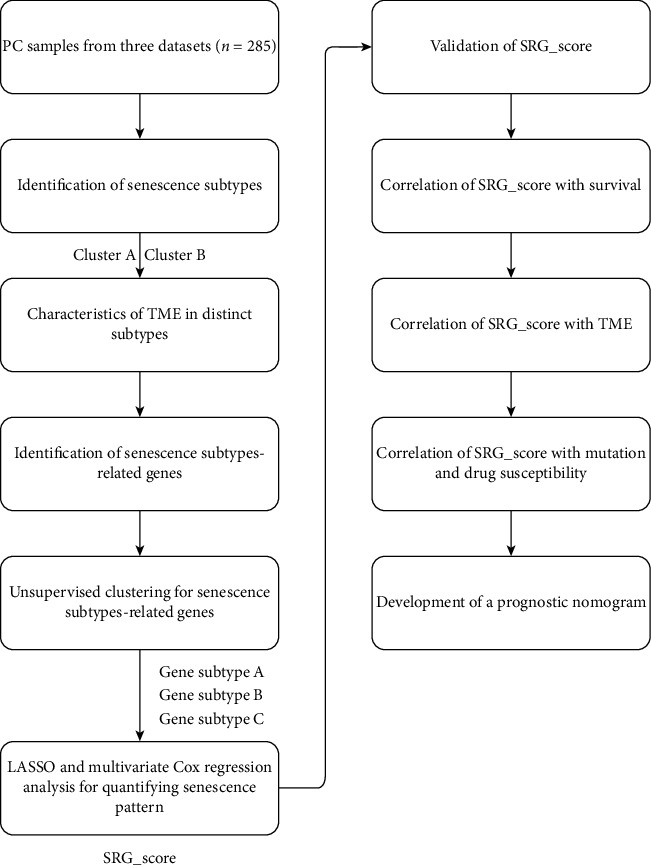
Flow diagram of the study design and analysis of the senescence-related genes in pancreatic cancer patients.

**Figure 2:**
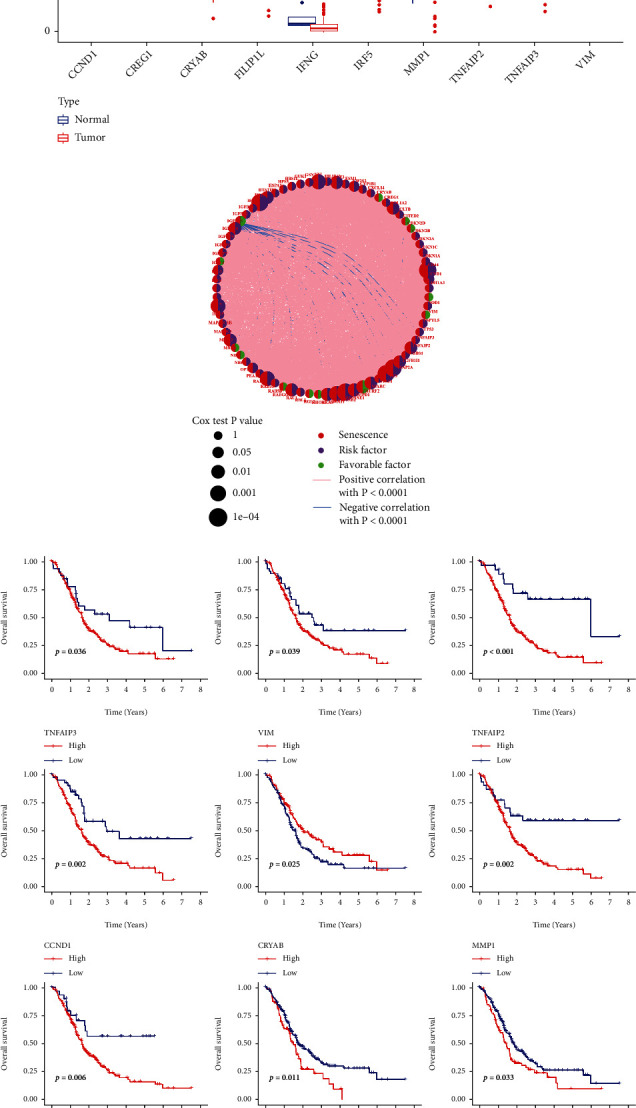
Expressions of senescence-related genes in pancreatic cancer and normal tissues. (a) Differences in the mRNA expression values of the ten senescence-related genes between normal and pancreatic cancer samples. (b) Interaction of the senescence-related genes in PC. Lines connecting the senescence-related genes represent their interaction with each other, with the line thickness indicating the strength of the association between SRGs. Blue and pink represent negative and positive correlations, respectively. The size of each circle represents the prognostic effect of each regulator and scaled by the *p* value. (c) The Kaplan-Meier curve analysis of the senescence-related DEGs. The cohort was divided into two groups (high and low risk) based on the median risk score, which was used as the cutoff value. ^∗^*p* < 0.05, ^∗∗^*p* < 0.01, ^∗∗∗^*p* < 0.001, and ^∗∗∗∗^*p* < 0.0001; ns: not statistically significant; PC: pancreatic cancer; DEG: differentially expression gene.

The detailed process in this study is shown in Figure 1. A total of 77 SRGs were included in this study. Comprehensive dissection of the expression level of these genes between some PC samples and some adjacent nontumor samples from TCGA was conducted. The results showed a relatively high expression level of CCND1 and MMP1 in the PC samples. While the expression level of CREG1, CRYAB, FILIP1L, IFNG, IRF5, TNFAIP2, TNFAIP3, and VIM in the PC tissues exhibited significantly lower than that in the normal pancreatic tissues (Mann-Whitney *U* test, ^∗^*p* < 0.05; ^∗∗^*p* < 0.01; ^∗∗∗^*p* < 0.001; *p* ≥ 0.05, not significant) (Figure 2(a)). Subsequently, we constructed a comprehensive network to deeply investigate the connection and mutual function of the senescence genes in the PC samples (Figure 2(b)). Survival analysis revealed that expression levels of CCND1, MMP1, CREG1, CRYAB, FILIP1L, IFNG, TNFAIP2, TNFAIP3, and VIM had an impact on the prognosis of PC patients with *p* values < 0.05 (Figure 2(c)). The results demonstrated that these specific genes influenced the development of PC and patients' survival.

### 3.2. Identification of Senescence Clusters in PC

**Figure 3:**
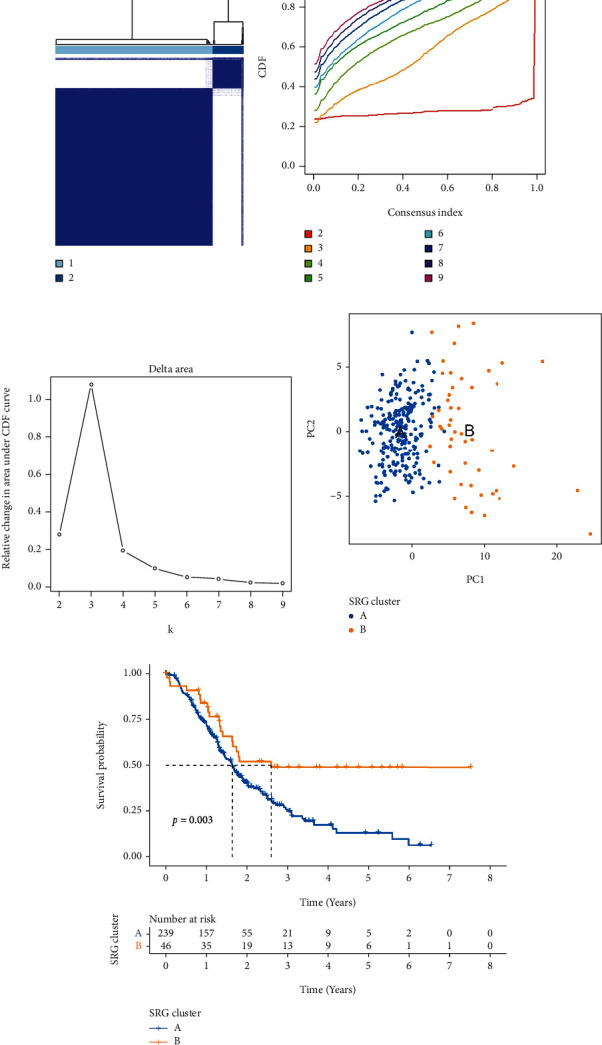
Two distinct SRG clusters of samples divided by consistent clustering. (a–c) Consensus matrix heatmap defining two clusters (*k* = 2) and their correlation area. (d) PCA analysis showed a remarkable difference in transcriptomes between the two clusters. (e) The Kaplan-Meier curves showed significant survival differences between cluster A and cluster B. SRG: senescence-related gene; PCA: principal components analysis.

To better understand the potential biological molecule of SRG related to tumorigenesis, we integrated three eligible PC cohorts (TCGA, GSE57495, and GSE62452) correlated with follow-up in our study for further analysis. After removing the normal pancreatic tissues, the prognostic values of 77 PRGs with *p* < 0.05 were selected as the threshold for filtering according to the univariate Cox regression and Kaplan-Meier analyses. To further investigate the expression characteristics of SRGs in PC, we applied unsupervised clustering methods to categorize the patients with PC into different molecular subgroups. By gradually adding the clustering variable (*k*) from 2 to 9, we identified *k* = 2 as the optimal cluster number to divide the entire cohort into cluster A (*n* = 239) and B (*n* = 46) using a consensus clustering algorithm (Figures 3(a)–3(c)). According to principal component analysis (PCA), we observed that there was a significant difference between the two clusters (Figure 3(d)). The Kaplan-Meier curves showed a longer OS in patients with cluster B than that in patients with cluster A (log-rank test, *p* = 0.003; Figure 3(e)).

### 3.3. Characteristics of the TME in Distinct Subtypes

**Figure 4:**
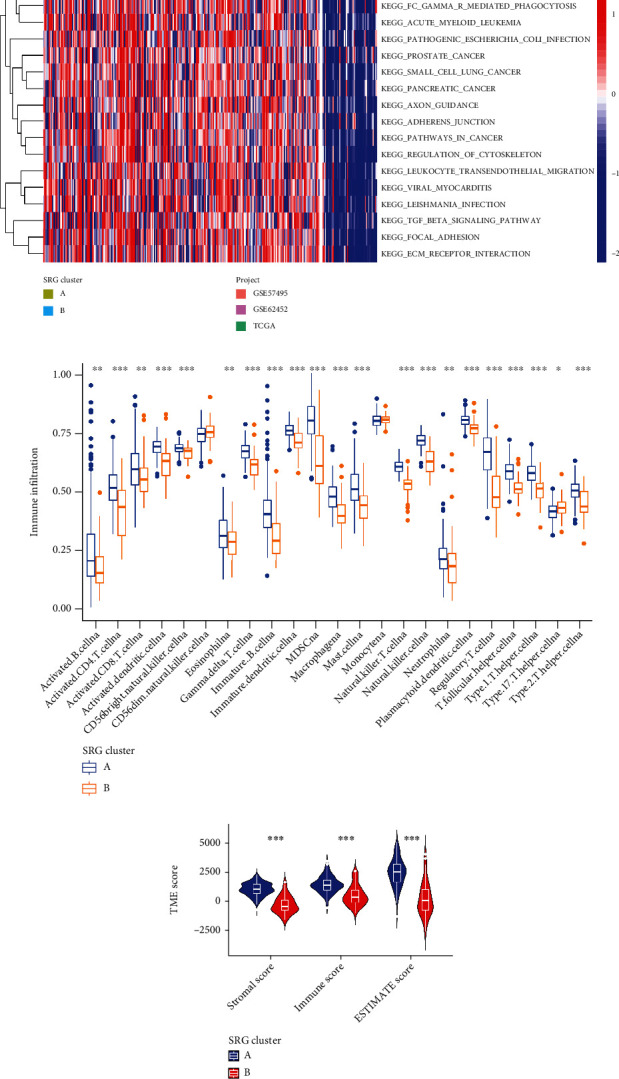
Correlations of tumor immune cell microenvironments and two PC clusters. (a) GSVA of biological pathways between two distinct clusters, in which red and blue represent activated and inhibited pathways, respectively. (b) Abundance of 23 infiltrating immune cell types in the two clusters. (c) Correlations between the two clusters and TME score. PC: pancreatic cancer; GSVA: gene set variation analysis; TME: tumor microenvironment.

To investigate the biological characteristics of these distinct molecular clusters, GSVA enrichment analysis was performed, and the results showed that cluster A was not only significantly enriched in immune-related pathways, including Fc gamma R-mediated phagocytosis, transforming growth factor beta (TGF-*β*) signaling pathway, regulation of actin cytoskeleton, focal adhesion, extracellular matrix (ECM) receptor interaction, and adherens junction, but also in cancer-related pathways, such as pancreatic cancer, prostate cancer, renal cell carcinoma, chronic myeloid leukemia, and acute myeloid 1 (Figure 4(a)). To confirm whether SRGs are associated with the TME of PC, we compared the human immune cell enrichment scores between cluster A and cluster B using the CIBERSORT algorithm. It was found that the infiltration levels of most immune cells were obviously higher in the cluster A than those in the cluster B (Figure 4(b)). Meanwhile, in the assessment of the TME score, including stromal score, immune score, and estimate score, we utilized the R “estimate” package to explore the immune-related score between the two subtypes. The results demonstrated that the patients with cluster A have higher TME score (Figure 4(c)).

### 3.4. Function Enrichment Analysis and Identification of Gene Subtypes Based on DEGs

**Figure 5:**
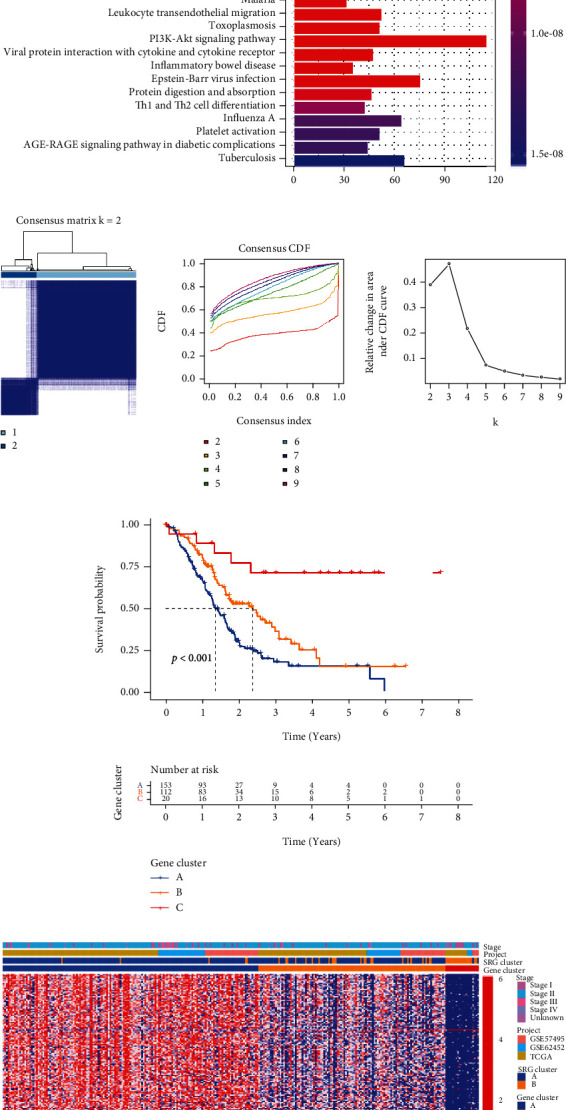
Identification of gene subtypes based on DEGs. (a, b) GO and KEGG enrichment analyses of DEGs among two senescence clusters. (c) Unsupervised consensus clustering identified three independent subclusters based on the expression levels of the differentially expressed genes. (d) The Kaplan-Meier curves for OS of the three gene subtypes (log-rank tests, *p* < 0.001). (e) Relationships among clinicopathologic feature, the two clusters, and the three gene subtypes. (f) Differences in the expression of SRGs among the three gene subtypes. DEGs: differentially expressed genes; GO: Gene Ontology; KEGG: Kyoto Encyclopedia of Genes and Genomes; OS: overall survival; SRGs: senescence-related genes.

We identified DEGs between the two subtypes using the R package “limma” and performed functional enrichment analysis, including GO and KEGG enrichment analyses. The differentially expressed genes between the cluster A and the cluster B were partially expressed at high levels in immune-related biological processes (Figure 5(a)). The consequence of the KEGG pathway analysis indicated that the differentially expressed genes also were significantly enriched in pathways pertained to immune aspect (Figure 5(b)), demonstrating that senescence can be regarded as a pivotal role in the immune regulation of the TME. Next, we used a consensus clustering algorithm to categorize patients into gene subtypes A–C based on prognostic DEGs (Figure 5(c)); the Kaplan-Meier curves showed that patients with gene subtype A had the worst OS, whereas patients in gene cluster C showed a favorable OS (log-rank test, *p* < 0.0001; Figure 5(d)). And the different expression patterns of these genes in the two clusters, three gene subtypes, and clinicopathological feature are depicted in a heatmap (Figure 5(e)). The box plot showed the prominent differences in the mRNA expressions of these genes among the three gene subtypes (Figure 5(f)).

### 3.5. Construction of the Prognostic SRG_score

**Figure 6:**
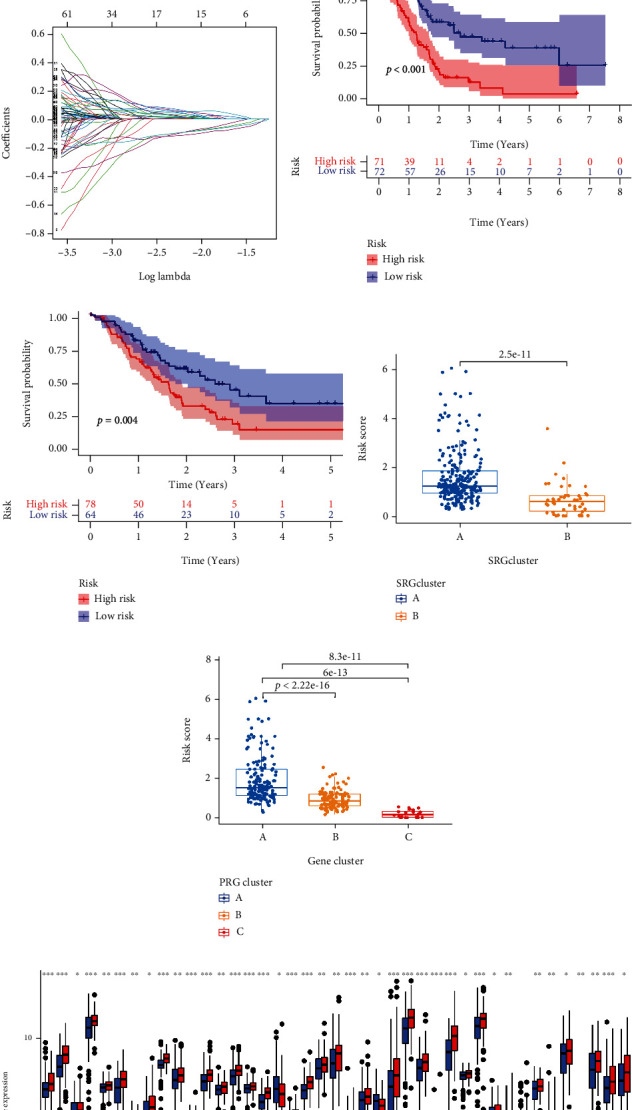
Construction of the SRG_score in the training set. (a) Alluvial diagram of cluster distribution in groups with different SRG_scores and survival outcomes. (b, c) The LASSO Cox regression model was constructed from the prognostic genes, and the tuning parameter (*λ*) was calculated based on the partial likelihood deviance with 10-fold cross-validation. An optimal log *λ* value is indicated by the vertical black line in the plot. (d) The Kaplan-Meier analysis of the prognosis between the two groups in training cohort. (e) The Kaplan-Meier analysis of the prognosis between the two groups in testing cohort. (f) Differences in SRG_score between senescence subtypes. (g) Differences in SRG_score between gene subtypes. (h) Expression of senescence-related DEGs in the high- and low-risk groups. SRG: senescence-related gene; DEGs: differentially expressed genes.

The SRG_score was established based on the subtype related DEGs.  Figure 6(a) illustrates the distribution of patients in the two senescence clusters, three gene subtypes, and two SRG_score groups. First, we used the “caret package” in R to randomly classify the patients into training (*n* = 143) and testing (*n* = 142) groups at a ratio of 1 : 1. LASSO-penalized multivariate Cox analyses were performed to further select optimum prognostic signature. Finally, a prognostic signature comprising five genes, including TRPS1, KCNH3, CDA, ATP1A3, and FLRT3, was developed according to the minimum partial likelihood deviance (Figures 6(b) and 6(c)). A novel risk score was calculated by multiplying the expression of each gene and its corresponding coefficient, which was obtained by multivariate Cox regression analysis. SRG_score = (0.4339 × expression value of TRPS1) + (−0.3478 × expression value of KCNH3) + (0.1856 × expression value of CDA) + (−0.4464 × expression value of ATP1A3) + (0.2314 × expression value of FLRT3). We divided PC patients into high and low SRG score groups based on the median value. Survival analysis suggested that high-risk patients had a significantly worse prognosis than that in patients with low scores in training cohort (log-rank test, *p* < 0.001; Figure 6(d)) and testing cohort (log-rank test, *p* = 0.004; Figure 6(e)). The distributions of risk scores in the two clusters and three gene subtypes are shown in Figures 6(f) and 6(g). The Kaplan-Meier analysis demonstrated that the five included senescence-related genes are dependent of their roles in the prognosis of PC patients with *p* values < 0.05 (Figure S1). There were differences in the expression level of senescence-related DEGs between the high-risk group and the low-risk group (Figure 6(h)).

**Figure 7:**
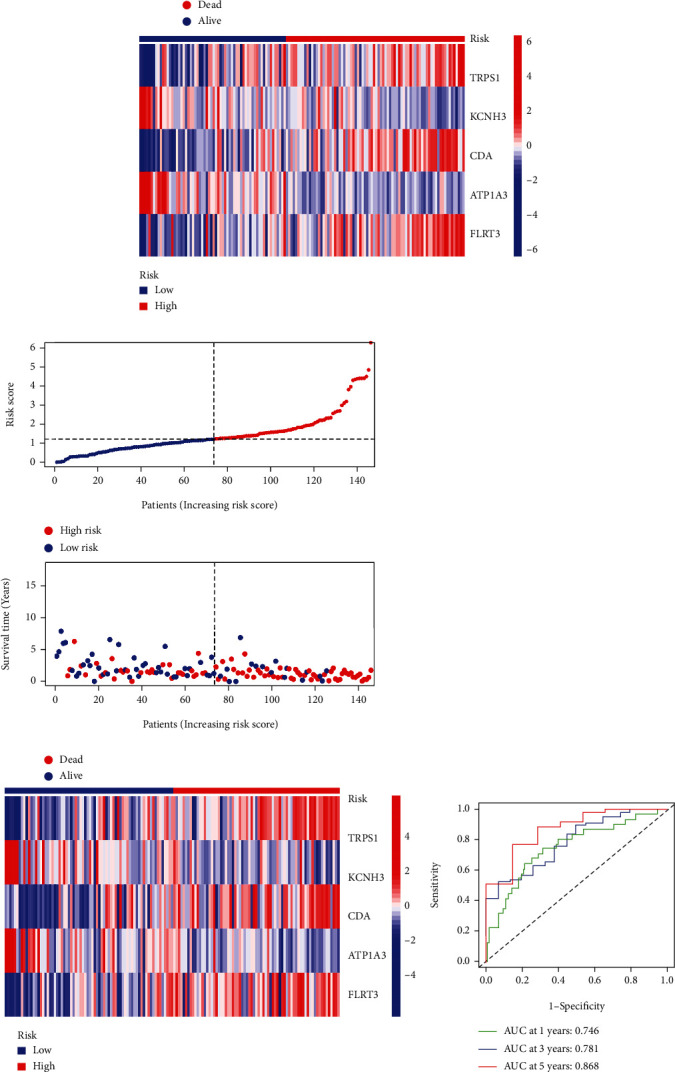
The risk survival status chart of PC cohort. (a) The risk survival status plot of the patient in the training cohort and the number of patients who died increased with the increase in patient risk score. (b) The risk survival status plot of the patient in the testing set. (c) ROC curves to predict the sensitivity and specificity of 1-, 3-, and 5-year survival in the training set and testing set according to the SRG_score. PC: pancreatic cancer; ROC: receiver operating characteristic.

In both sets of cohorts, the distribution of the risk scores and vital statuses of patients was the same (Figures 7(a) and 7(b)). In addition, to estimate the predictive performance of this model, time-dependent ROC curves were used to estimate the validity of the five SR risk assessment tool constructions in the two cohorts, and the area under time-dependent ROC curve (AUC) values was calculated at 0.746, 0.781, and 0.868 representing the 1-, 3-, and 5-year survival rates of SRG_score in the training group, respectively (Figure 7(c)). Similarly, the AUCs were equal to 0.653 at 1 years, 0.755 at 3 years, and 0.785 at 5 years in the testing group (Figure 7(d)). All the AUC values were more than 0.6, which implied that the model could achieve satisfactory predictive accuracy in the two cohorts.

### 3.6. Evaluation of TME between the High- and Low-Risk Groups

**Figure 8:**
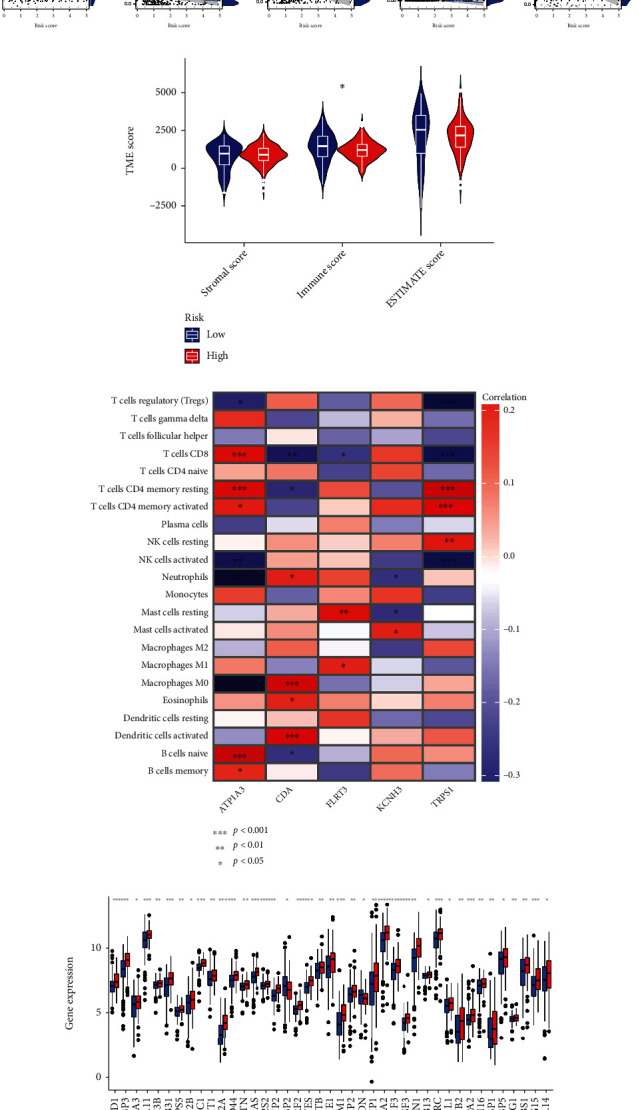
Evaluation of the TME between the two groups. (a) Correlations between SRG_score and immune cell types. (b) Correlations between SRG_score and both immune and stromal scores. (c) Correlations between the abundance of immune cells and five genes in the proposed model. (d) Expression of senescence-related genes in the high- and low-risk groups. TME: tumor microenvironment; SRG: senescence-related gene.

We performed the CIBERSORT algorithm to investigate the correlation between the SRG_score and the tumor immune cell infiltration. The results of Spearman's test showed that the SRG_score was positively linked with M0 macrophages, dendritic cells activated, M2 macrophages, mast cell resting, and neutrophils and negatively correlated with B cells memory, T cell CD4 memory resting, CD8+ T cells, naive B cells, and monocytes (Figure 8(a)). A low SRG_score tended toward a higher degree of immune score, while although there is no difference between the SRG_score and stromal score, a stromal score was higher in low SRG_score patients (Figure 8(b)). We also assessed the association between tumor immunity and the five genes in the proposed model. We found that several infiltrating/immune cells were obviously correlated with the five genes (Figure 8(c)).

### 3.7. Mutation and Drug Susceptibility Analysis

Given TMB has been identified as a critical role in the development of tumor, we explored the differences in the distribution of somatic mutations between high- and low-risk groups.

**Figure 9:**
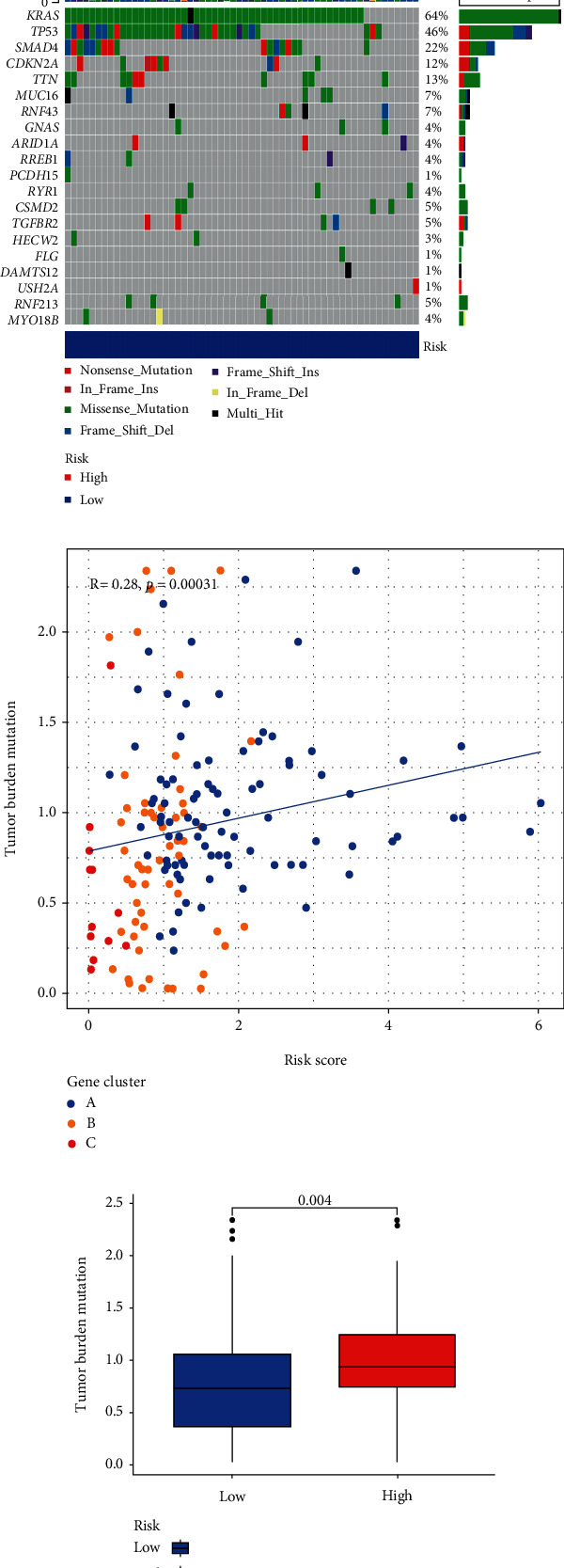
Comprehensive analysis of the mutation and drug susceptibility of the SRG_score in PC. (a, b) The waterfall plot of somatic mutation features established with high and low SRG_scores. Each column represented an individual patient. The upper bar plot showed TMB, and the number on the right indicated the mutation frequency in each gene. The right bar plot showed the proportion of each variant type. (c) Spearman correlation analysis of the SRG_score and the three gene subtypes. (d) TMB in different SRG_score groups. (e) Relationships between SRG_score and chemotherapeutic sensitivity. PC: pancreatic cancer; TMB: tumor mutation burden.

The top 20 most frequently mutated genes of these two groups were shown in Figures 9(a) and 9(b), respectively. Missense mutation was the most common among all mutation types, and KRAS had the highest mutation frequency. Spearman's correlation analysis indicated that the SRG_score elevated with the increase of TMB in the gene subtypes (*R* = 0.28, *p* = 0.00031; Figure 9(c)). The TMB score was lower in the low-risk group compared to the high-risk group (Figure 9(d)). We next investigated an association between the sensitivities of chemotherapy drugs currently used for the treatment of PC and the SRG_score through the Genomics of Drugs Sensitivity in Cancer (GDSC) database. We found that the patients in the high SRG_score group were linked to lower IC_50_ value for gemcitabine, while the patients with low SRG_score were linked to lower IC_50_ values of chemotherapeutics such as axitinib. Together, these results showed that PRGs were related to drug sensitivity (Figure 9(e)).

### 3.8. Development of a Nomogram to Predict Survival

**Figure 10:**
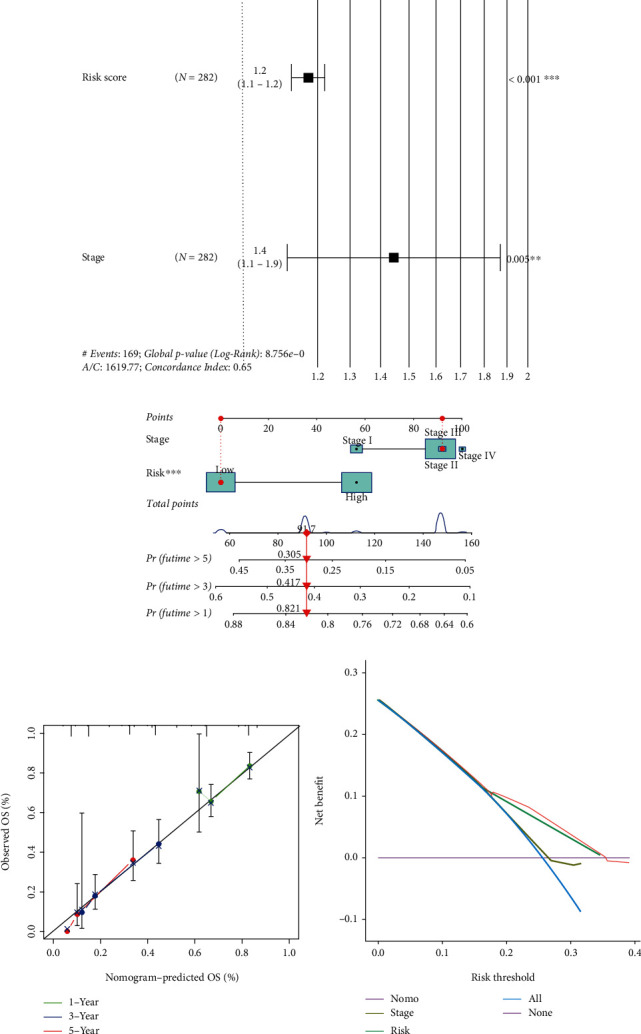
Development of a nomogram in the training set. (a) Forest plot of the multivariate Cox regression analysis for stage and SRG_score. (b) Nomogram for predicting the 1-, 3-, and 5-year OS of PC patients in the training set. (c) Calibration curves of the nomogram for predicting the 1-, 3-, and 5-year OS. (d) DCA analysis compared the nomogram with SRG_score and stage, respectively. SRG: senescence-related gene; OS: overall survival; DCA: decision curve analysis.

On the basis of the multivariate Cox analysis, the forest plot revealed that both stage and SRG_score were independent risk factors for OS of PC patients (Figure 10(a)). To establish a quantitative approach for OC prognosis, we integrated the SRG_score and independent clinical risk feature to construct a nomogram (Figure 10(b)). The total score was utilized to predict the 1-, 3-, and 5-year OS of the PC patients. Moreover, the decision curve analysis (DCA) verified that the nomogram showed superiority in predicting the OS compared with SRG_score and stage, respectively (Figure 10(c)). Calibration curves for the probability of OS at 1, 3, and 5 years showed that there is a satisfactory consistency between actual observation and nomogram-predicted OS probabilities in the PC cohort (Figure 10(d)).

## 4. Discussion

Pancreatic cancer is one of most common malignancies of the digestive system with extremely low 5-year survival rate [24]. Only a few patients are eligible for resection due to an advanced stage at the time of diagnosis [25]. Beyond that, pancreatic cancer is also not sensitive to additional treatments, including radiation, chemotherapy, and immune checkpoint inhibitor-based (ICI-based) immunotherapy, because of immunosuppressive and desmoplastic microenvironment [26]. Consequently, better understanding of the molecular mechanism of PC might have a fundamental impact on the treatment response. The advent of programmed cell death, an active death process, has considerably improved the stability of the internal environment not only in the normal development of individuals but also in abnormal physiological conditions or diseases [27]. Senescence tends to occur during the earliest stages of PC [28]. It exerts an indispensable function in tumor development by secretion of senescence-associated secretory phenotype (SASP) [29]. However, the synergistic effects of various senescence-related genes have not yet been fully elucidated in PC. In the present study, we revealed two distinct molecular subtypes based on the expression of 77 SRGs. We further analyzed the prognosis and immune cell infiltration condition between the two subtypes. Patients with cluster B were characterized by a better OS, lower immune scores, and less immune cell infiltration compared to patients with subtype A. According to the functional enrichment analysis, antigen processing, and presentation, Fc gamma R-mediated phagocytosis, TGF-*β* signaling pathway, regulation of actin cytoskeleton, focal adhesion, ECM receptor interaction, and adherens junction were exhibited in SR-A tumors, which were related to immune aspect. Thus, it is found that SRGs might serve as potential diagnostic or therapeutic targets for assessing the clinical outcome and immunotherapy response of PC. To explore the molecular differences among different subtypes, we further identified the DEGs of the two subtypes. A total of DEGs were determined to be associated with the important prognostic value of PC. After screening by the univariate Cox regression analysis, log-rank test, and LASSO Cox method, 5 genes were ultimately regarded to construct the stable and effective prognostic SRG_score and validate its predictive ability, including TRPS1, KCNH3, CDA, ATP1A3, and FLRT3, which was proved to be efficient by survival and ROC analysis. These genes had been reported to be related with other cancers and might be potential novel prognostic factors of PC [30, 31]. In this study, the SRG score could link senescence and prognosis and showed good performance in predicting the survival of patients. In addition, for the sake of the facility of clinical application, a nomogram was produced including risk score and stage, which were practical and easy to offer more utility risk stratification to distinguish the patients with markedly distinct survival outcomes.

Our results also highlight that prognosis, TME, and drug susceptibility differed significantly between patients with high SRG_score and those with low SRG_score. It has been reported that the inability of immune cells in the tumor microenvironment led solid cancers to escape from host immunity, which indicated that the immune microenvironment plays a key role in the occurrence and development of cancer [32–35]. These findings represent a new insight to improve discussions on patient prognostication and stratification through considering the microenvironment characteristics and transcriptomics. Thus, the immune condition and senescence correlation with their interaction in tumor microenvironment and those relating to PC progression could bring us to enter an era of discussion with PC with respect to prognosis. In the present study, we found that subtype A was linked to a higher SRG_score while subtype B could indicate a lower SRG_score, which implied that the immune level had an important implication in prognostic outcome.

Nowadays, cancer was considered to be a heterogeneous disease not only relating to abnormal mutations in tumor cells but also resulting from their microenvironmental component and stromal cell proportions or activation states [36, 37]. Notably, the two major groups of cellular and noncellular elements in TME are important for tumorigenesis and tumor types. Besides, the distinct characteristic of TME responsible for tumor develops had an essentially effect on tumor growth, metastasis, and prognosis [38, 39]. TME is mostly made up of nonmalignant cells of the tumor such as immune cells, granulocytes, lymphocytes, and macrophages, which engage in a variety of immune responses and activities, and the ECM establishes a sophisticated link with tumor [40]. Stromal densification, composed of 200 different cellular and noncellular compositions, is a pronounced histological feature of pancreatic cancer and is also regarded as desmoplastic reaction or TME. During the last 10-15 years, emerging clinical and preclinical studies supported the pivotal role of TME in pancreatic tumorigenesis [41]. Regarding anticancer, previous studies also underscored that senescence plays a vital role in improving advanced cancer patients' clinical outcomes and prognoses by regulating the TME [42, 43]. We discovered that the characteristics of the TME and the relative abundance of 23 TIICs differed significantly between the two molecular subtypes and different SRG_scores. This finding suggests the crucial effect of SRGs in PC progression.

In this study, we systematically investigated the TME immune cell infiltration level in different two heterogeneous senescence-related subgroups (A and B). It was found that the A subgroup possessed a higher content of immune cells than that of the B subtype. B cells are beneficial for the prognosis of cancer patients due to the effective suppression of tumorigenicity [44, 45]. Low-risk group has tended toward higher B cell infiltration levels than those in high-risk group. In addition, high-risk group was also notable for the B cell naïve, which plays a master role in promoting tumor. Neutrophils are inclined to enhance PC development and progression [46]. Tumor-infiltrating B cells were also related to a favorable prognosis for PC. Meanwhile, given the pivotal effect of immune cells in the transformed pancreas, various methods of stimulating T cell activity and their antitumor capacity have been explored [47]. Our observation found that more CD8+ T cells, monocytes, and T cell CD4 memory resting were infiltrated in the TME of the low-risk group. These findings supported the point that senescence score was significantly associated with overall survival and patients in the low senescence score group exhibited a superior prognosis. Increasing evidence showed the importance of macrophages in the pathogenesis of PC via influencing T cell-mediated tumor function based on macrophage phenotype [48]. Moreover, the M2 phenotype of tumor-associated macrophages clustering into the stroma is immunosuppressive for cancer patients and promotes cancer progression [49, 50]. High tumor stromal density of M2 macrophages predicts worse prognosis in cancer patients and accelerates the metastasis of cancer [51–53]. Next, we further found that patients with low SRG_score group possessed lower macrophages M2 level, implying that the SRG_score holds important value for TME. A high TMB means more favorable immunotherapy due to a great deal of neoantigens [54]. Based on our analysis, SRG_score presented a significantly negative correlation with TMB, indicating that patients with a high SRG score may provide more profitable outcomes for immunotherapy.

With the in-depth study of adjuvant chemotherapy, appropriate selection of chemotherapy drugs is conducive to improve the prognosis of advanced cancer patients [55]. Gemcitabine has been suggested as the first-line therapy for patients with advanced PC and improve quality of life [56, 57]. Axitinib could function as a selective inhibitor of VEGF receptors 1, 2, and 3 [58], and the result of phase I study of axitinib in combination with gemcitabine indicated that this combination showed performance in encouraging antitumor activity [59]. Cisplatin is beneficial for metastatic in combination with gemcitabine [60]. Inhibition of AKT improved the anticancer cell proliferation, migration, and invasion [61]. In this study, SRG_score also played an important role in affecting the sensitivity of PC to chemotherapy. It was found in this study that the two subgroups had significantly distinctive drug sensitivity in terms of several anticancer drugs.

By exploring the estimate IC_50_, patients in the low-risk subgroup showed superiority in sensitizing to axitinib, lenalidomide, metformin, methotrexate, vorinostat, and temsirolimus as compared with those in the high-risk subgroup, while patients in the high-risk subgroup may gain more benefit from gemcitabine, cisplatin, bortezomib, dasatinib, pazopanib, and cytarabine. According to the results of risk score, different patients can obtain more effectively sensitive chemotherapy drugs single or in combination, which more conformed to the opinion of individualized treatment in precision medicine.

This study had several limitations. First, this research was conducted solely on data from the TCGA and GEO public databases. Therefore, additional in vivo and in vitro experimental studies will be conducted to confirm our findings. Furthermore, data on some important clinical variables such as neoadjuvant chemotherapy and chemoradiotherapy were unavailable for analysis in most datasets, which resulted in the need for clinical trials.

## 5. Conclusions

This study expanded the knowledge about the function of TME in tumor progression, drug sensitivity, and prognostic value of SRGs in PC. We also identified the therapeutic responsibility of SRGs in PC. These findings highlight the crucial clinical implications of SRGs and provide innovative strategy for guiding individualized precise therapy for patients with PC.

## Data Availability

The datasets analyzed during the current study are available from the corresponding author on reasonable request.

## Abbreviations

TIICs: Tumor-infiltrating immune cells
TCGA: The Cancer Genome Atlas
GEO: Gene Expression Omnibus
SRG: Senescence-related gene
DEGs: Differentially expressed genes
ROC: Receiver operating characteristic
FPKM: Fragments per kilobase million
TPM: Transcripts per kilobase million
GSVA: Gene set variation analysis
TMB: Tumor mutation burden
ICIs: Immune checkpoint inhibitors
ssGSEA: Single-sample gene set enrichment analysis
PCA: Principal component analysis
AIC: Akaike information criterion
TME: Tumor microenvironment
PC: Pancreatic cancer
SASP: Senescence-associated secretory phenotype
TGF-*β*: Transforming growth factor beta
ECM: Extracellular matrix
GDSC: Drug sensitivity in cancer
IC_50_: Semi-inhibitory concentration
GO: Gene Ontology
KEGG: Kyoto Encyclopedia of Genes and Genomes.

## Supplementary Materials

Supplementary Materials Figure S1: the Kaplan-Meier curve analysis of the five senescence-related genes used for the construction of the SRG_score.
