# Supplementary figures and images for: The Senescence-Related Signature Predicts Prognosis and Characterization of Tumor Microenvironment Infiltration in Pancreatic Cancer
Source: Biomed Res Int. 2022 Dec 5;2022:1916787. doi: 10.1155/2022/1916787 (PMC9744624; doi:10.1155/2022/1916787)

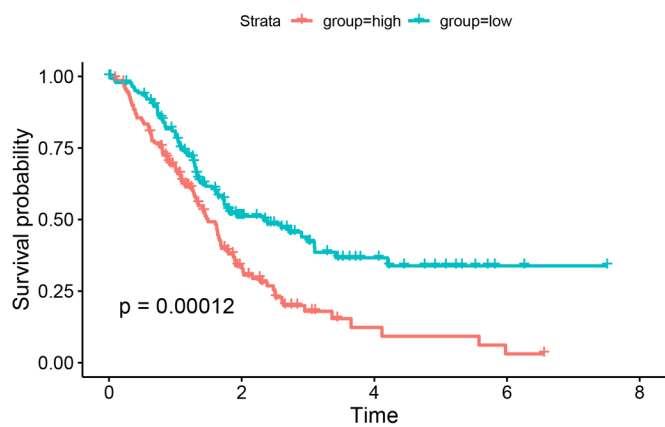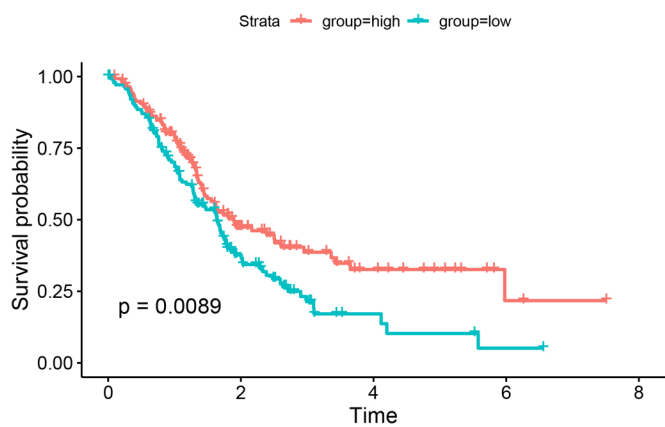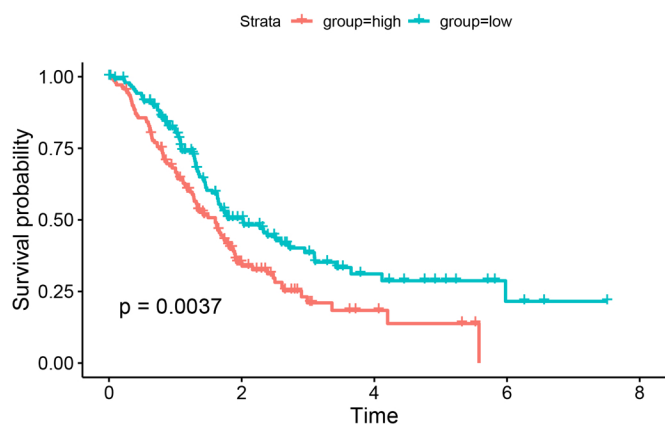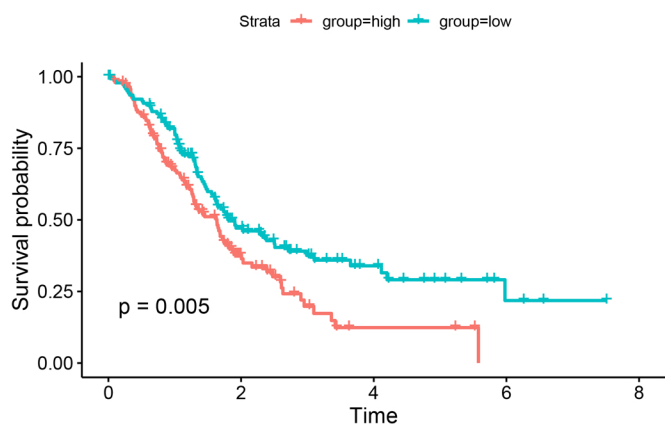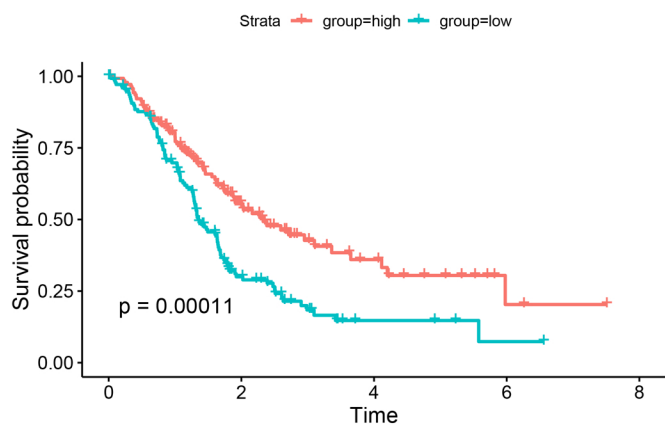

Supplement: Supplementary Materials — Figure S1: the Kaplan-Meier curve analysis of the five senescence-related genes used for the construction of the SRG_score. [file 1916787.f1.pdf]
